# progressiveMauve: Multiple Genome Alignment with Gene Gain, Loss and Rearrangement

**DOI:** 10.1371/journal.pone.0011147

**Published:** 2010-06-25

**Authors:** Aaron E. Darling, Bob Mau, Nicole T. Perna

**Affiliations:** 1 Genome Center and Department of Computer Science, University of Wisconsin, Madison, Wisconsin, United States of America; 2 Biotechnology Center and Department of Oncology, University of Wisconsin, Madison, Wisconsin, United States of America; 3 Genome Center and Department of Genetics, University of Wisconsin, Madison, Wisconsin, United States of America; University of California Riverside, United States of America

## Abstract

**Background:**

Multiple genome alignment remains a challenging problem. Effects of recombination including rearrangement, segmental duplication, gain, and loss can create a mosaic pattern of homology even among closely related organisms.

**Methodology/Principal Findings:**

We describe a new method to align two or more genomes that have undergone rearrangements due to recombination and substantial amounts of segmental gain and loss (flux). We demonstrate that the new method can accurately align regions conserved in some, but not all, of the genomes, an important case not handled by our previous work. The method uses a novel alignment objective score called a sum-of-pairs breakpoint score, which facilitates accurate detection of rearrangement breakpoints when genomes have unequal gene content. We also apply a probabilistic alignment filtering method to remove erroneous alignments of unrelated sequences, which are commonly observed in other genome alignment methods. We describe new metrics for quantifying genome alignment accuracy which measure the quality of rearrangement breakpoint predictions and indel predictions. The new genome alignment algorithm demonstrates high accuracy in situations where genomes have undergone biologically feasible amounts of genome rearrangement, segmental gain and loss. We apply the new algorithm to a set of 23 genomes from the genera *Escherichia*, *Shigella*, and *Salmonella*. Analysis of whole-genome multiple alignments allows us to extend the previously defined concepts of core- and pan-genomes to include not only annotated genes, but also non-coding regions with potential regulatory roles. The 23 enterobacteria have an estimated core-genome of 2.46Mbp conserved among all taxa and a pan-genome of 15.2Mbp. We document substantial population-level variability among these organisms driven by segmental gain and loss. Interestingly, much variability lies in intergenic regions, suggesting that the Enterobacteriacae may exhibit regulatory divergence.

**Conclusions:**

The multiple genome alignments generated by our software provide a platform for comparative genomic and population genomic studies. Free, open-source software implementing the described genome alignment approach is available from http://gel.ahabs.wisc.edu/mauve.

## Introduction

Multiple genome alignment is among the most basic tools in the comparative genomics toolbox, however its application has been hampered by concerns of accuracy and practicality [1]–[3]. Accurate genome alignment represents a necessary prerequisite for myriad comparative genomic analyses.

During the course of evolution, genomes undergo both local and large-scale mutational processes. Local mutations affect only a small number of nucleotides and include nucleotide substitution and insertion or deletion of nucleotides. Large-scale mutations can include gain and loss or duplication of large segments, generated by unequal recombination or other processes. Homologous recombination can lead to replacement of whole genes, or even larger segments of the chromosome with non-identical but homologous sequences. Together, these mutational processes cause otherwise identical regions in two or more genomes to be fragmented, reordered, possibly missing, and even to occur in multiple copies.

The genome alignment task seeks to identify the homologous nucleotides in two or more genomes, that is, a genome alignment identifies nucleotides that descended from a single site in some ancestral organism. Homologous sites can be classified in any number of ways, and the genome alignment task usually targets the identification of certain classes of nucleotides. Homologous sites are commonly classified by evolutionary history such as orthology, paralogy, and xenology [4], [5]. Sites can also be classified by non-evolutionary relationships such as the number or identity of organisms involved (e.g. only homologous sites involving an important reference organism such as *Homo sapiens*), or even by ordering relationships relative to other homologous nucleotides (e.g. collinearity). Genome alignment methods often define their target alignment to consist of homologous nucleotides falling into one or more of those classes.

Early work in genome alignment included development of MUMmer, which identifies homologous sites in pairs of genomes [6]–[8]. MUMmer aligns orthologous and xenologous sequences with the further constraint that any site in a genome can be aligned to at most one site in the other genome. Pairs of homologous sites within a single genome (paralogs) are never aligned to each other. The first stage of MUMmer alignment involves identifying alignment anchors. Alignment anchors are local alignments of highly identical sequence that by virtue of their high identity, can be easily found algorithmically, and are presumed to be part of the true alignment. MUMmer then aggregates local alignment anchors into one or more groups that cover collinear regions of the two genomes. Each group of anchors is internally free from rearrangement, but the order of groups may be shuffled from one genome to another. As such, MUMmer can identify and align genomes with rearranged homologous sequences. However MUMmer does not align paralogous sequences (repeats within a genome), nor does it align all copies of multi-copy orthologous sequence. Because it aligns any site to at most one site in the other genome, and due to the way it anchors alignment of repetitive sequence using neighboring unique regions, MUMmer often aligns the positionally conserved copy of a repeat element. We term this type of alignment a *positional homology genome alignment*; such alignments are also generated by a method we developed previously [9].

In the present work, we describe a new method to construct *positional homology multiple genome alignments* that extends our previous method [9] to aligning regions conserved in subsets of the genomes. The new method can align a larger number of genomes than the previous method, and does so with higher accuracy as demonstrated by simulation. The previous method has especially low sensitivity in regions conserved among some but not all organisms, whereas the new method can align those same differentially conserved regions with high accuracy. Three algorithmic innovations factor strongly in our method's ability to align genomes with variable gene content and rearrangement. The first is a novel objective function, called a sum-of-pairs breakpoint score, to score possible configurations of alignment anchors across multiple genomes. Our second algorithmic contribution is a greedy heuristic to optimize a set of anchors under the sum-of-pairs breakpoint score. Finally, we demonstrate that most anchored alignment techniques suffer a bias leading to erroneous alignment of unrelated sequence in regions containing differential gene content. Our third algorithmic contribution is the application of a homology hidden Markov model (HMM) to reject such erroneous alignments of unrelated sequence. The new method is implemented in a program called progressiveMauve, part of the Mauve genome alignment package versions 2.0 and later.

We compare the accuracy of alignment methods existing at the time of this work and the new alignment method on datasets simulated to encompass a broad range of genomic mutation types and rates, including inversion, gene gain, loss, and duplication. We then apply the multiple genome alignment method to a group of 23 finished genomes in the family Enterobacteriacae (Table S1). We precisely identify the core- and pan- genomes of this group independently of annotated gene boundaries, and report basic analysis of gene flux patterns in Enterobacteriacae. Development of our new alignment algorithm was inspired by genomic studies of *E. coli*, which revealed substantial gene content variability among individual *E. coli* isolates [10], [11]. Since those early studies, gene content variability has been reported as a common feature in numerous other microbial species [12]–[15]. It appears that microbial populations undergo vast amounts of gene gain, loss and homologous recombination [16], although most systematic studies have been limited to gene-based methods by the difficulty of complete and accurate multiple genome alignment. Our aligner offers a platform on which to base study of the combined effects of gene gain, loss, and rearrangement in microbial species.

### Previous genome alignment methods

Approaches to whole-genome alignment typically reduce the alignment search space using anchoring heuristics [17]–[22] or banded dynamic programming [23]. Anchoring heuristics appear to provide a good tradeoff between speed and sensitivity. Most anchored alignment methods assume that the input sequences are free from genomic rearrangement. As such, a separate synteny mapping algorithm must be applied to map collinear homologous segments among two or more genomes prior to alignment. Synteny mapping approaches are too numerous to list, however most involve computing reciprocal best BLAST hits on putative ORFs, with BLAST hits filtered by e-value thresholds, coverage thresholds, and uniqueness criteria. Some synteny mapping methods apply genomic context to help resolve ambiguous orthology/paralogy relationships, and others use probabilistic transitive homology approaches to infer homologs among distantly related taxa [24].

Integrated approaches to synteny mapping and alignment have been proposed, most of which operate on pairs of genomes [8], [25]–[27]. Research into multiple alignment with rearrangements has been limited, although some progress has been made [9], [28]–[31]. Apart from greater ease-of-use, integrated synteny mapping and alignment methods could in theory provide more accurate inference because the alignment can influence the synteny map and vice-versa.

**Figure 1:**
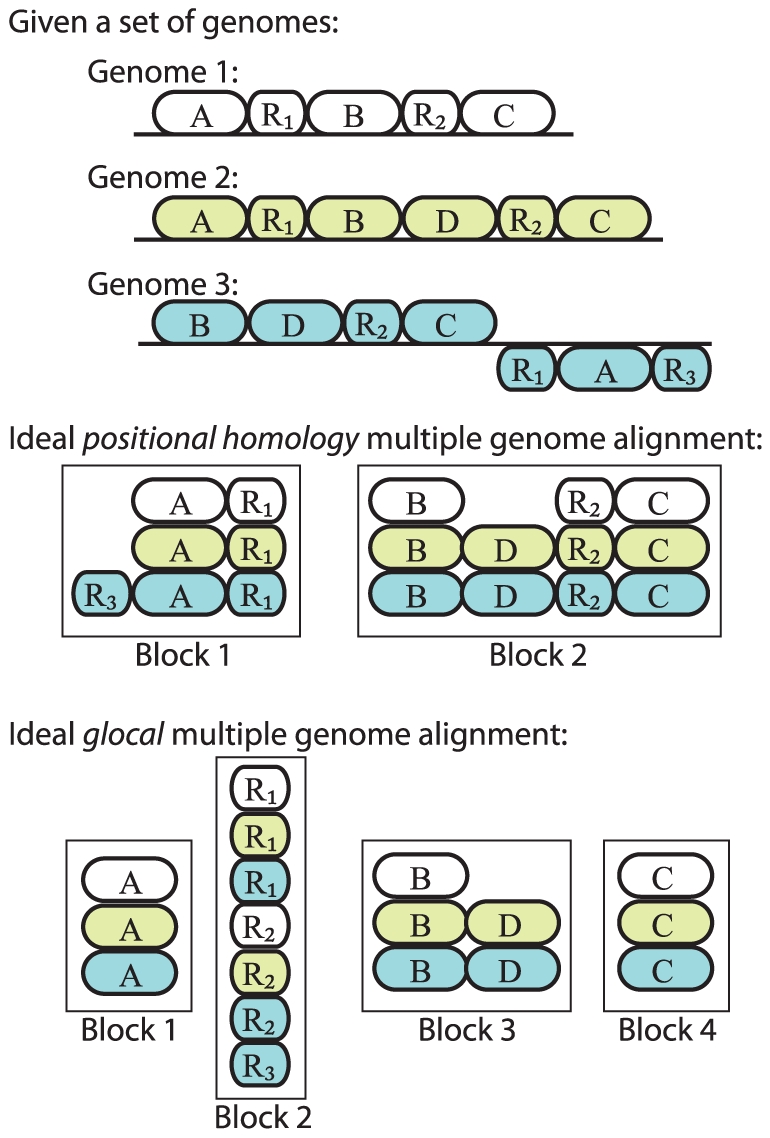
The difference between *positional homology* alignment and *glocal* alignment. Three example linear genomes are broken into genes labeled A,B,C,D, and R. R is a multi-copy (repetitive) gene, with different copies labeled using numeric subscripts. Each copy of R is assumed to be identical in sequence, so that orthology/paralogy is unknowable from nucleotide substitution (as is often the case with mobile DNA repeat elements). Genes shifted downward in a given genome are inverted (reverse complement) relative to the reference genome. The *positional homology* alignment would ideally create two local alignment blocks where each block has exactly one alignment row for each genome. Only positionally-conserved copies of the repetitive gene family R become aligned to each other. The *glocal* alignment would ideally create four local alignment blocks wherein all copies of the repetitive gene family become aligned to each other.

New methods for genome alignment have become available since the time of this work. Two of these methods construct so-called *glocal* multiple genome alignments [32], [33] (see [25] for a definition of glocal). The main distinguishing feature in how those methods align genomes lies in how they handle repetitive segments. Instead of aligning the positionally conserved copy of a repetitive DNA segment (*a la* Mauve), *glocal* methods construct a multiple alignment of all homologous copies of the repetitive segment, regardless of whether they are orthologous or paralogous. Figure 1 illustrates the difference using three example genomes. We note that by concatenating several genomes into a single sequence, methods for large-scale local multiple alignment of genomic DNA [34], [35] can also be used to compute glocal alignments, requiring only a “de-concatenation” step after alignment. The task of identifying the positionally homologous region and subclassifying homology relationships into types of orthology or paralogy is left for downstream inference methods. Such an approach has advantages when applied to duplication-rich metazoan and plant genomes, for which positional homology is often not as clear as in smaller microbial genomes. In organisms with clear positional homology, however, the need to resolve duplication histories in *glocal* alignments can unecessarily complicate downstream inference tasks when compared to positional homology alignments. As discussed below, a large number of tools exist to analyze positional homology alignments that can not be applied to *glocal* alignments.

## Methods

**Figure 2:**
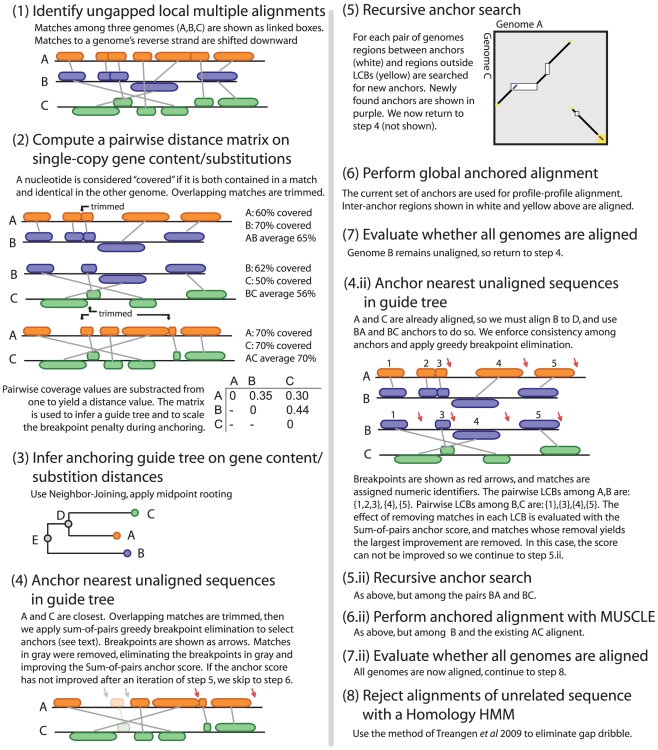
Overview of the alignment algorithm using three example genomes A, B, and C.

An overview of our method as applied to three hypothetical genomes appears in Figure 2 and is presently described in detail.

### Notation and assumptions

Our genome alignment algorithm takes as input a set of 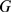 genome sequences 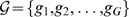. We denote the length of genome 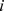 as 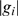. Contigs in unfinished or multi-chromosome genomes are concatenated to form a single coordinate system. Various default parameter settings in our software implementation depend on the average length of the input genome sequences, which we denote by 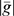. Genomic coordinates are assumed to start at 1, and increase in magnitude from left to right. Coordinates can be denoted by a signed integer 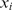 in 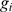. The sign of 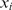 indicates strandedness, with negative values denoting alignments to the reverse strand. Let 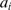 be the corresponding nucleotide base at genomic position 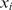; when 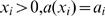, and when 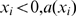 is the complementary base of position 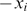. Finally, 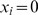 indicates a gap in genome 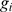. The basic building blocks of the whole genome alignment are local multiple alignments (LMAs), which we will denote by 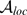. We use LMAs that generalize MUMmer's Maximal-Unique-Matches (MUMs) to include approximate matching and multiple genomes.

### Local Multiple Alignments as potential anchors

We identify local multiple alignments as potential anchors using families of palindromic spaced seed patterns [36] in a seed-and-extend hashing method (see Appendix of [9]). A spaced seed pattern of length 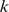 and weight 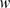 [37] identifies the location of 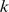-mers in the input genomes that have identical nucleotide sequence except that a small number of mutations are allowed at fixed positions. For example, the seed pattern 11*11*11 would identify matching oligomers of length 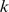 = 8 where the 3rd and 6th positions are degenerate. The number of 1's in the seed pattern is commonly referred to as the weight of the seed pattern, denoted 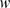. Thus the pattern 11*11*11 has 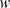 = 6. A pattern is said to be palindromic if the pattern is identical when read forward or in reverse [38]. A seed family is a collection of seed patterns that when used in conjunction provide improved matching sensitivity, and such families have been previously demonstrated to offer excellent speed and sensitivity [39].

To minimize compute time and focus anchoring coverage on single-copy regions, our method only extends seeds that are unique in two or more genomes. By default, we use seed patterns with weight equal to 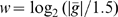. This formula is also applied to determine the appropriate seed weight during recursive anchoring (Figure 2 step 5, described later), with the restriction that 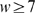 in all cases. The resulting local multiple alignments are ungapped and always align a contiguous subsequence of two or more genomes in 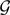. Any given local multiple alignment 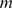 can be described formally by its length 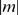 and vector of integers: 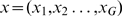, where 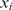 is a signed left-end coordinate of the LMA in 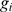, or 0. When 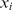 takes on a value of 0, the 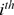 genome is absent from all of 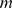.

The LMAs found by our procedure are ungapped alignments of unique subsequences and thus are similar to multi-MUMs, but may contain mismatches according to the palindromic seed patterns. As with multi-MUMs, any portion of a unique LMA may be non-unique and no LMA may be completely contained within the boundaries of another LMA. We refer to the set of local multiple alignments generated in this step as 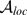. An example is given in Figure 2 step 1.

### Local alignment anchor scoring

Given a pairwise alignment without gaps in genomes 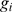 and 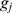, we compute a pairwise substitution score using a substitution matrix, which defaults to the HOXD matrix [40]. The HOXD matrix appears to discriminate well between homologous and unrelated sequence in a variety of organisms, even at high levels of sequence divergence.

The substitution matrix score quantifies the log-odds ratio that a pair of nucleotides share common ancestry, but does not account for the inherent repetitive nature of genomic sequence. Our desire to discriminate between alignment anchors that suggest positional homology and alignments of regions with random similarity or paralogy requires that we somehow consider repetitive genomic sequence in our anchoring score [41].

We combine the traditional substitution score for a pair of nucleotides with an adjustment for the multiplicity of 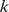-mer seeds at the aligned positions:
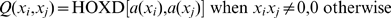
(1)

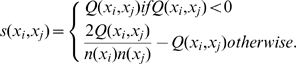
(2)where 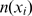 is the number of occurrences of the spaced seed pattern that matches the subsequence of 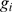 at 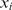. The product of 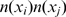 approximates the number of possible ways that sites in 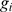 and 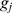 with the same seeded 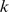-mers as 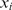 and 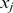 could be combined. For example, consider a repeat element present in both genomes with copy number 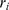 in genome 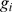 and copy number 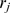 in 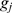. There are 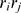 possible pairs of repeats. When a pair of nucleotides in a repeat element have a positive substitution score, the product 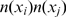 down-weights the score.

In summary, this scoring scheme assigns high scores to well-conserved regions that are unique in each genome and does not consider gap penalties.

### Pairwise locally collinear blocks

A pair of genomes 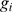 and 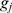 may have undergone numerous genomic rearrangements since their most recent common ancestor. As such, local alignments among orthologous segments of 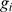 and 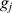 may align segments that occur in a different order or orientation in each genome. We define a pairwise locally collinear block (LCB) as a subset of local alignments in 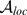 that occur in the same order and orientation in a pair of genomes 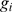 and 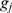 they are free from internal rearrangement. To define pairwise LCBs among genomes 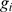 and 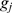, we first define the projection of the current set of local multiple alignments 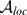 onto 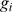 and 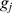 as 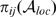, realized by setting all coordinates for genomes 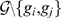 to 0. In local alignment 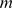 for example, the projection 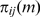 onto 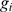 and 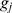 is obtained by setting all left-end coordinates: 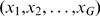 to 0 except for 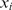 and 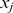.

Having transformed LMAs into local pairwise alignments, we apply the well-known breakpoint analysis procedure [42], [43] to minimally partition 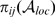 into pairwise LCBs. Let 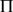 denote the minimal partition of a projection 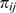 into disjoint LCBs: 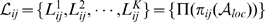. Projecting onto two dimensions allows us to apply the previously described scoring scheme.
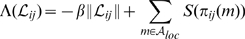
(3)where 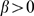 is a fixed constant, 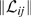 is the number of pairwise LCBs formed in the projection of 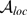 onto 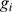 and 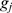, and
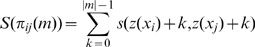
(4)

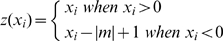
(5)therefore 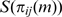 computes the sum of scores for each pair of sites in 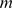 that are aligned in genomes 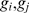. The function 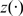 computes the matching sequence coordinates in matches that contain reverse-complement regions.

**Figure 3:**
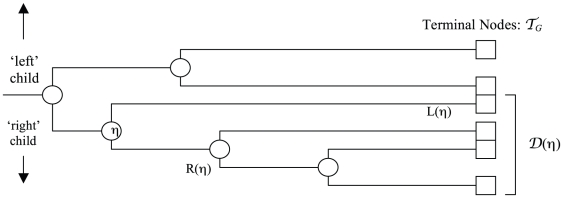
Illustration of terminology used for tree nodes. Rotating the figure 90 degrees counterclockwise explains the descriptive use of left and right.

Our method computes alignments along a rooted guide tree 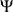. We use 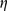 to denote an arbitrary internal node of 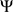, and the set of leaf (or terminal) nodes by 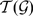. As 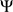 is a rooted bifurcating tree, each internal node 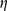 has two children, designated by 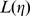 and 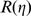 for left and right child. The terms left and right are for notational convenience, and have no intrinsic meaning. We denote the set of leaf nodes descended from 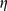 as 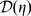. This terminology is illustrated in Figure 3.

We compute the following sum-of-pairs LCB anchoring (SP anchoring) objective function to select a set of alignment anchors:
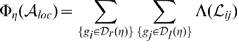
(6)along a guide tree whose construction is described below.

### Anchoring guide tree construction

Our method computes alignment anchors progressively according to a guide tree computed as follows. We compute a genome-content distance matrix and Neighbor Joining tree based on the initial set of local multiple alignments 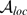. Values in the genome-content distance matrix are computed as described in Figure 2 step 2. Information about shared genomic content factors strongly into the distance metric, so that organisms with similar genomic content tend to cluster. The topology of the resulting guide tree may not represent the clonal genealogy of the organisms, however, we have found that guide trees based on genomic content allow our algorithm to produce better alignments. We also note that users may change the guide tree to one of their own choosing via a command-line option. The resulting tree is midpoint-rooted to yield our progressive anchoring guide tree.

The guide tree is used for anchoring but not for the gapped global multiple sequence alignment, wherein alignments are optimized over a variety of guide trees muscle [44]. Steps 2 and 3 in Figure 2 illustrate guide tree construction.

### Optimizing the SP anchoring objective function

In equation 3, the constant 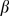 is a breakpoint penalty, and when multiplied by 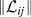, creates a scoring penalty that increases in magnitude when the anchors in 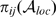 induce a larger number of LCBs. Thus, anchor sets inducing fewer breakpoints are given higher scores.

Recent versions of the algorithm apply a genome-pair-specific scaling of the breakpoint penalty 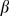, based on the expected divergence among the organisms in terms of genomic content and rearrangements. The scaling is motivated by the biological phenomenon of rate heterogeneity in evolutionary processes. The rates of gene gain, loss, rearrangement, and nucleotide substitution appear to vary independently of one another across lineages, and the scaling factor helps to account for this fact. We omit mathematical derivation of the scaling here for brevity and clarity of presentation.

The value of the breakpoint penalty 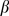 is a user controlled parameter in our implementation of the algorithm, and we use a default minimum scaled value of 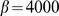 as manual experimentation on real genome sequence data suggests this value represents a good tradeoff between sensitivity to small genomic rearrangements and filtration of spurious alignments. When 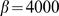, a rearranged segment as short as 40nt may be aligned, so long as it is perfectly identical and in single-copy in both genomes. We arrive at that figure by observing that the highest score for a nucleotide match in the HOXD matrix is 100, so that 40 consecutive matching nucleotides would have a score of 4000 according to equation 4, but only if all 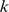-mer seeds in that region are unique. The minimum scaled value of 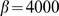 was selected by testing increasing values of 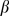 on genomes with 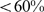 nucleotide identity until a value was found that excluded most spurious alignments, as determined by BLAST and gene annotation.

We apply a greedy breakpoint elimination heuristic to optimize 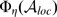 which removes potential anchors from 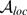 until the score can no longer be increased. Removing the matches which constitute a single LCB 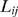 decreases the total number of LCBs in 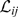 by at least one and at most four if neighboring LCBs coalesce [9]. The number of LCBs in projections to other genome pairs may decrease as well. The decreased number of LCBs, and hence breakpoints, reduces the total breakpoint penalty in 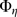. But the anchoring function has two components, and 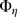 increases only if 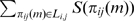 has a sufficiently small total score, favouring the deletion of “small” LCBs that “interrupt” large LCBs.

Our algorithm iteratively identifies the LCB whose removal from 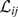 would provide the largest increase to 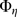. This procedure corresponds to step 4 in Figure 2. Formally, we identify the 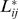 that maximizes:
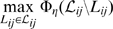
(7)over 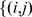, where 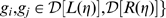.

When deleting one 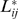 from the current set of pairwise LCBs 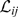, we simultaneously remove those local alignments 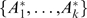 whose projections generate 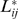. Therefore removal of a pairwise LCB from 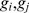 may simultaneously remove LCBs and breakpoints from other pairwise projections. Multiple iterations of the optimization procedure result in a strictly decreasing sequence of LMAs: 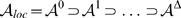.

The greedy breakpoint elimination process repeats until further removal of LCBs (and their constituent LMAs) fails to improve the SP anchoring score at node 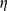. The procedure is repeated successively at each of the 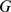-1 internal nodes of the guide tree 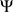, starting with the two nearest genomes in 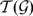 and proceeding through the guide tree to the root.

### Recursive anchoring

The initial set of local alignments in 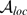 is typically computed using a seed weight that finds local alignments in unique regions of high sequence identity 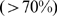. As such, the initial set of anchors frequently misses homologous regions with lower sequence identity. After anchor selection by greedy breakpoint elimination (Equation 4), our method searches for additional local alignments between anchors existing among all pairs of genomes in 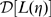 and 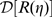, see Figure 2 step 5.

To improve sensitivity during recursive anchor search, smaller seed weights are used as described by [9]. Any new local alignments are added to 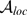. Consistency is enforced among the new anchors and they are merged to form multi-genome anchors. After the recursive anchor search, we apply greedy breakpoint elimination to optimize the SP anchor score once again. The recursive anchoring and breakpoint elimination steps repeat until 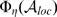 no longer improves by more than 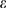 percent. The value of epsilon defaults to 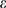 = 0.5%. This limit prevents the aligner from expending large amounts of computational effort to improve the anchoring by a trivial amount.

### Anchored profile alignment and iterative refinement

The alignment anchors 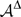 computed at node 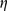 are used to perform an anchored profile-profile global alignment with modified MUSCLE 3.7 software [44]. Global profile-profile alignment requires the input sequences to be free from rearrangement. Therefore, we partition the anchors in 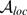 into groups that are free from breakpoints in any pairwise projection. A fully fledged locally collinear block 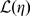 at node 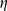, no longer constrained to two dimensions, is a maximal set 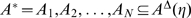 in which each pair-wise projection of 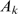 into 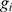 and 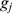 in 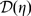 is contained in a common pair-wise LCB in 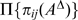. One or more of the original pair-wise LCBs from 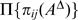 may be truncated by this restriction, and hence the partitioning into LCBs at node 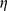 can be thought of as the intersection among constituent pairwise LCBs. Then each LCB in 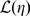 is independently subjected to anchored profile-profile alignment using methods described elsewhere [44]. In order to capture the full region of homology at the boundaries of each LCB, sequence regions outside LCBs are randomly split and assigned to neighboring LCBs. An example is shown with the yellow regions in Figure 2 step 5.

After the initial profile-profile alignment, we then apply window-based iterative refinement to improve the alignment. Step 6 of Figure 2 corresponds to this process. Importantly, MUSCLE refines the alignment with a multitude of alternative guide trees and is not restricted to the guide tree chosen for progressive anchoring. The use of multiple guide trees is a particularly important feature in microbial genomes, which are subject to lateral gene transfer. It should be noted that our use of MUSCLE as a refinement step is an approach used in other software pipelines as well [45].

### Rejecting alignments of unrelated sequences

Segments of DNA between high-scoring alignment anchors can be unrelated, especially in bacteria. Despite that, our method (like many other genome aligners) applies a global alignment algorithm to all inter-anchor segments, navely assuming that homology exists. Our assumption of homology sometimes proves erroneous, so to arrive at an accurate alignment we must detect forced alignment of unrelated sequence. To do so, we apply an HMM posterior decoder that classifies columns in a pairwise alignment as either homologous or unrelated. The HMM structure, transition, and emission probabilities are described elsewhere [34]. The HMM makes predictions of pairwise homology, which we combine using transitive homology relationships. Regions found to be unrelated are removed from the final alignment. Application of the homology HMM is the final step in the alignment procedure, shown as step 8 in Figure 2.

### Implementation

The alignment algorithm has been implemented in the progressiveMauve program included with Mauve v2.0 and later. The program is open source C++ code (GPL), with 32- and 64-bit binaries for Windows, Linux, and Mac OS X available from http://gel.ahabs.wisc.edu/mauve. An accessory visualization program is included. Default alignment parameters have been calibrated for bacterial genomes [38].

## Results

### Quantifying alignment accuracy

Our new alignment algorithm uses approximations and computational heuristics to compute alignments. To understand the quality of alignments produced by our approach it is essential to objectively quantify alignment accuracy. Without a known ‘correct’ genome alignment, automated alignment heuristics can not be evaluated for accuracy. Although several benchmark data sets exist for protein sequence alignment [44],[46], no such benchmark data sets exist for genome alignment with rearrangement. Thus far, manual curation of a megabase-scale whole-genome multiple alignment that includes rearrangement and lateral gene transfer has proven too time-consuming and difficult. Despite the lack of a manually curated correct alignment, we can estimate the alignment accuracy by modeling evolution and aligning simulated data sets. All results described in this section and the programs used to generate them are available as supplementary material.

### Simulated evolution model

In previous work, we constructed a genome evolution simulator that captures the major types, patterns, and frequencies of mutation events in the genomes of Enterobacteriacae [9]. We use the same simulated model of evolution in the present study but with different evolutionary parameters. Given a rooted phylogenetic tree and an ancestral sequence we generate evolved sequences for each internal and leaf node of the tree, along with a multiple sequence alignment of regions conserved throughout the simulated evolution. Along the branches, mutations such as nucleotide substitution, indels, gene gain/loss, and inversion rearrangements are modeled as a marked Poisson process. We score calculated alignments against the correct alignments generated during the evolution process.

Although gene duplication occurs very frequently in bacteria, we do not *explicitly* model it here as duplications tend to be unstable in bacterial chromosomes and are often counterselected [47]. That is, duplications generally do not persist for long periods of time. Instead, we indirectly model gene duplications in two ways. First, the source DNA sequence for gene gain events comes from a 1Mbp pool of sequence. At moderate to high simulated rates of gene gain, many megabases of DNA are sampled from the donor pool, and as a result, identical donor sequence gets inserted into the simulated genomes in multiple places. The effect is similar to a dispersed repeat family, such as bacterial IS elements or mammalian SINE elements.

Second, we use the genome sequence of *E. coli* O157:H7 as ancestral sequence and as donor sequence for all insertion and gene gain events. The *E. coli* O157:H7 genome has numerous naturally occurring repeats that are carried on to simulated descendant genomes, and is among the largest of the sequenced *E. coli* genomes, providing as much natural starting material for simulation as possible. By using real genome sequence as ancestral sequence, the resulting evolved genomes often have similar nucleotide, dinucleotide, 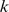-mer composition, repeat copy number and repeat distribution. The unknown natural forces governing the evolution of such traits would otherwise be extremely difficult to capture in a simulation environment.

Our experimental results at high mutation rates should be interpreted with caution, however, since the more simulated mutations applied, the less a simulated genome will look like a real genome. This is a shortcoming of all forward-time evolution simulations and we are unaware of any solution to this problem. Nevertheless, simulation studies remain the only practical way to objectively measure the quality of multiple genome alignments.

### Accuracy evaluation metrics

Previous studies of alignment accuracy have used a sum-of-pairs scoring scheme to characterize the residue level accuracy of the aligner [9], [46]. The experiments presented here use sum-of-pairs scoring, but we also define new accuracy measures to quantify each alignment system's ability to predict indels and breakpoints of genomic rearrangement. For each type of mutation, we define True Positive (TP), False Positive (FP), and False Negative (FN) predictions as discussed below. Using these definitions, we can measure the aligner's Sensitivity as 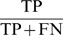 and Positive Predictive Value (PPV) as 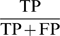.

For nucleotide pairs, a TP is a pair aligned in both the calculated and correct alignments. A FP is a nucleotide pair in the calculated alignment that is absent from the correct alignment. Likewise, a FN is a pair in the correct alignment not present in the calculated alignment. We do not quantify True Negative (TN) alignments as the number of TN possibilities is extremely large, growing with the product of sequence lengths.

**Figure 4:**
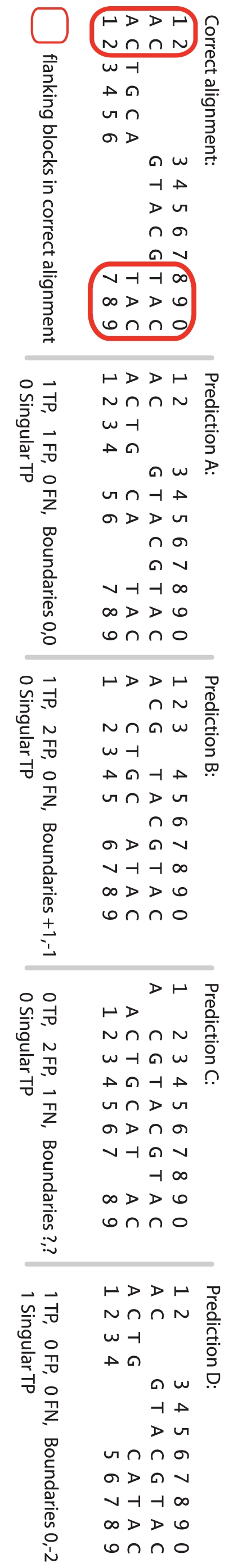
Quantifying indel accuracy. The correct alignment is shown at left and four possible predicted alignments are shown as A, B, C, and D. Nucleotides have been assigned a numerical identifier. The correct alignment has a single indel which partitions the alignment into three sections: the left aligned block, the indel, and the right aligned block. Predicted alignments must have one correctly aligned nucleotide pair in each of the three sections to count a true positive indel prediction.

We classify each indel in the correct alignment as a TP or a FN based on the predicted alignment. A true positive indel has at least one correctly aligned nucleotide pair in the diagonal/block on either side of the indel and at least one nucleotide correctly aligned to a gap within the indel (see Figure 4). The number of TP indels will never exceed the number of indels in the correct alignment. We define FP indel predictions as the number of excess indel predictions beyond the true positives. FN indels lack a correctly predicted nucleotide pair in the flanking diagonals/blocks or lack predictions of gaps in the correct gapped region. Figure 4 gives examples of each case.

Aligners are notoriously bad at predicting the exact position of indels [48]. Under our definition, a TP indel prediction need not predict the exact boundaries of an indel, merely the existence of an indel. This scheme allows us to distinguish cases of missing indel predictions from cases where the indel was predicted but not positioned correctly. We quantify indel boundary prediction accuracy as the distance between the true boundary and the nearest aligned nucleotide pair in the diagonal/blocks which flank the predicted indel. When the predicted indel is too large, our metric assigns a positive value to the boundary score. When the predicted indel is too small, a negative value is assigned.

Large indels have historically caused problems for nucleotide aligners, which have a tendency to break up large indels into a string of smaller gaps with intermittent aligned sequence. Under our definition, a large indel can still be considered as a TP prediction even if it is broken into a string of smaller gaps by the aligner (See Figure 4 prediction A for an example). Our rationale is that the aligner did correctly predict the presence of unrelated sequence, for which it garners a TP, but erroneously predicts additional transitions to and from homology, which are classified as FP indel predictions. To distinguish whether a TP indel was broken into two or more smaller gaps, we define a class of “singular” TP indel predictions as indels that were predicted as a single alignment gap. See Figure 4 prediction D for an example of a “singular” TP indel.

### Sum-of-pairs LCB accuracy and breakpoint localization

For each pair of genomes we also measure whether the aligner correctly predicts LCBs among that pair, yielding a sum-of-pairs LCB accuracy metric. For each pairwise LCB in the true alignment, we record a TP LCB prediction when the predicted alignment contains at least one correctly aligned nucleotide pair in that LCB. Pairwise LCBs lacking any correctly predicted nucleotide pairs are FN predictions. Finally, pairwise LCBs in the predicted alignment lacking any correctly aligned nucleotide pairs are False Positive (FP). Again, we do not measure TN.

As with indels, we define a separate metric to quantify how well each aligner localizes the exact breakpoints of rearrangement. For TP LCB predictions, we record the difference (in nucleotides) between the boundaries of the correct LCB and those of the predicted LCB. The resulting value is negative when the predicted LCB fails to include the full region of homology, and positive when a predicted LCB extends beyond the true boundary.

The rationale behind the LCB accuracy metrics is that they are robust to misprediction of LCB boundaries and effects induced by prediction of extra LCBs. For example, if a predicted LCB contains a single correctly aligned pair of nucleotides and is much shorter than the true LCB, then the error will be recorded as LCB boundary prediction error. In another example, imagine a single true LCB is split into two predicted LCBs with a third false positive LCB intervening. Our metric would record 1 True Positive and 1 False Positive. The LCB boundary scores would be determined by how far the left and right boundaries of the true LCB lie to the nearest boundaries of the predicted LCBs that have correctly aligned nucleotides in that true LCB. This approach prevents the False Positive LCB from disturbing our measurement of LCB boundary accuracy.

Under our definitions of TP, FP, TN, and FN predictions, specificity, which is commonly defined as 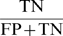, is not a useful metric. The extremely large values taken on by TN would drive the quotient to 1 in most cases.

### Selection of aligners for testing

We downloaded and tested all multiple-genome aligners that were publicly available as of May 2008, when this work was completed. Multi-genome aligners known to handle rearrangements at that point in time included mauveAligner 1.3.0 [9], progressiveMauve 2.2.0, and TBA 28-02-2006 [17]. We did not test two-stage pipelines involving separate synteny mapping and alignment steps, such as MERCATOR+MAVID [49] or Chain-net+TBA [50] but this would be an interesting area for future work. We did test a selection of available multiple-aligners that assume collinear genome sequences as input, including MLAGAN 2.0 [19], MAVID 2.0 [18], and Pecan 0.7 [33], which was available at the time of this work for download but not yet published. In the time since the aligner testing was completed, several new alignment systems have been published, including Enredo+Pecan [33], FSA [51], and an extension of LAGAN for reference-free alignment with duplication and rearrangements [32]. We did not test the duplication alignment accuracy of *glocal* alignment methods. Our simulation system does not explicitly model gene duplication, and worse, we do not know the true alignment of repeats in the ancestral genomic material, so it is impossible to quantify the accuracy of *glocal* aligners using our evaluation scheme. Testing of FSA [51] remains as future work. Finally, we did not test any of the numerous pairwise aligners or pairwise synteny mapping methods, as our work focuses on the multiple genome alignment problem.

Unless otherwise specified, we ran each aligner with default parameter settings. The aligners MLAGAN, TBA, and PECAN require specification of a guide tree for alignment; a guide tree is optional for MAVID. We supplied those aligners with the true simulation tree. mauveAligner and progressiveMauve were not supplied with the true simulation tree, but rather calculated their own guide tree for anchoring (a variety of local guide trees are used for alignment optimization). This potentially gives an advantage to MLAGAN, TBA, PECAN, and MAVID in the accuracy comparison, as in many cases of biological interest, a reasonable guide tree may not be identifiable prior to alignment. Three supplementary files described below contain complete command-line logs for each simulation and aligner run, along with the raw accuracy results.

### Accuracy on collinear genomes

**Figure 5:**
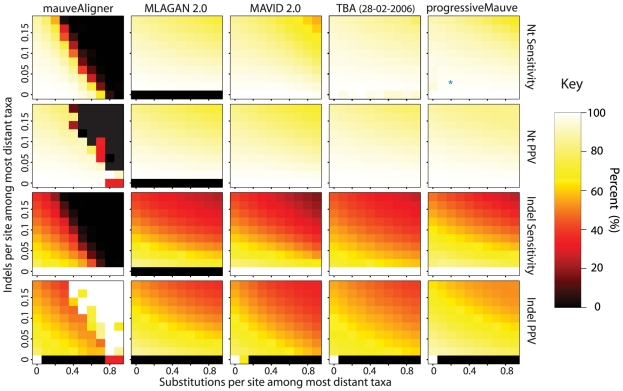
The accuracy of aligners on sequences evolved without rearrangement and with increasing nucleotide substitution and indel rates. Aligners were tested on 100 combinations of indel and substitution rate, with performance averaged over three replicates. All methods lose accuracy as mutation rates grow, and the most accurate alignment method depends on the particular mutation rates. progressiveMauve and MLAGAN exhibit the best indel sensitivity and positive predictive value (PPV), while TBA is more sensitive than other methods at extremely high mutation rates. MLAGAN did not align genomes without indels within the allotted 10 hours, resulting in the black row at the bottom. The asterisk in this figure indicates the combination of indel rate and substitution rate expected to be similar to our 23 target genomes.

**Figure 6:**
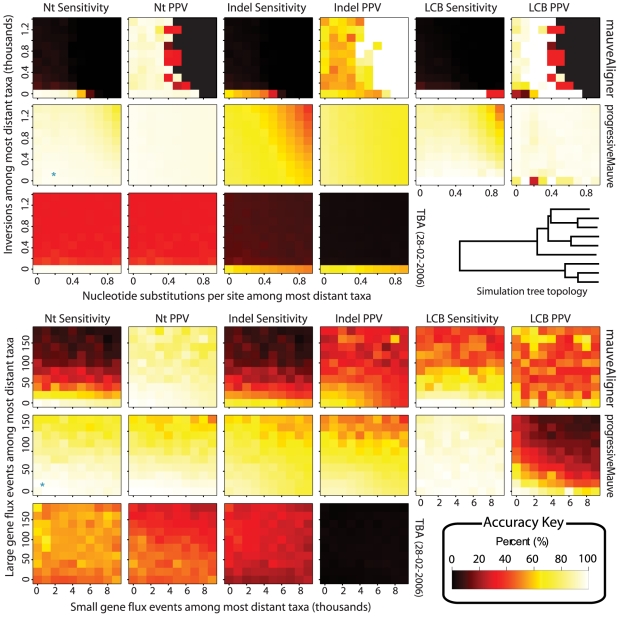
Accuracy of mauveAligner, progressiveMauve, and TBA when aligning genomes with inversions and segmental gain and loss. In the experiments shown at top, the inversion rate increases along the 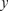-axis and the substitution rate along the 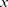-axis. The most distant taxa have 0.05 indels per site. progressiveMauve clearly outperforms mauveAligner 1.3.0 over the entire space of inversion rates. It should be noted that in applications such as the UCSC browser alignments TBA was used in conjuction with a separate synteny-mapping method to identify rearrangements [66], so the performance results given here are not cause for alarm. Experiments at bottom quantify aligner performance in the presence of small- and large-scale gain and loss events. The 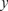-axis gives the average number of large gain and loss events [length
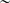
Unif(10kbp, 50kbp)] between the most distant taxa, while the 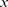-axis gives small gain and loss events [length
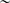
Geo(200bp)]. Substitution and indel rates are those indicated by the asterisk in Figure 5, and the most distant taxa have 42 inversions on average. The asterisk in this figure indicates a simulation scenario expected to be similar to our 23 target genomes. Once again progressiveMauve outperforms other methods, but all methods break down when faced with substantial large-scale gain and loss. Of note, when mauveAligner 1.3.0 attains high PPV it usually does so with very poor sensitivity.

Our first experiment compares the accuracy of mauveAligner 1.3.0, progressiveMauve, MLAGAN 2.0, MAVID 2.0, and TBA 28-02-2006 when aligning collinear sequences that have undergone increasing amounts of nucleotide substitution and indels. For each combination of indel and substitution rate, nine genomes are evolved from a 1Mbp ancestor according to a previously inferred phylogeny [9]. We then construct alignments of evolved sequences using each aligner with default parameters, and quantify sensitivity and positive predictive value, (PPV) for nucleotide pair and indel predictions. Three replicates were performed, results shown in Figure 5; the simulation tree is shown in Figure 6.

In general, all aligners perform well on collinear sequence, except for mauveAligner 1.3.0 which is unable to anchor genomes with high mutation rates. Of the tested aligners, TBA offers the highest nucleotide sensitivity, and progressiveMauve gives the best indel sensitivity and positive predictive value in most cases. Despite that, all aligners are quite bad at predicting indels accurately, which may be in part due to an inherent loss of information introduced during the course of simulated evolution [48]. We did not test the Pecan aligner here, although a detailed evaluation of its performance can be found elsewhere [45] and we do perform some testing on it below. We note that on the smaller set of simulated datasets for which we did test Pecan (below) it had higher indel sensitivity, nucleotide sensitivity, and nucleotide PPV than all other methods including progressiveMauve (data not shown).

The data corresponding to this simulation are available as File S1.

### Accuracy in the face of gain, loss, and rearrangement and gene

We assessed the relative performance of mauveAligner 1.3.0, progressiveMauve, and TBA [17] when aligning genomes with high rates of genomic rearrangement, gain, loss, and nucleotide substitution. Although the original TBA manuscript did not fully describe alignment with genomic rearrangement, the most recent release (dated 28-02-2006) handles it [28], [30]. For our first set of experiments, shown in the top half of Figure 6, we simulated evolution at 100 combinations of substitution and inversion rate. In addition to nucleotide and indel accuracy, we also quantify LCB accuracy on this data set. The results indicate that progressiveMauve can accurately align genomes with substantially higher rates of rearrangement than our previous approach. Although TBA exhibits lackluster performance on heavily rearranged genomes, comparison with the results for MAVID 2.0 and MLAGAN 2.0 (shown in Figure S1) demonstrates that for all rates of inversion, TBA produces much better alignments than methods which assume genomes are free from rearrangement. We are uncertain why TBA does not reach the same level of performance as progressiveMauve on heavily rearranged genomes, but discussion with the authors of TBA suggests that it may be a bug in the specific version available at the time of testing (Webb Miller, personal communication). A new version of TBA was available at the time of manuscript submission.

For the second set of experiments we simulated genomes with 10 increasing rates of small-scale segmental gain and loss and 10 increasing rates of large-scale segmental gain and loss. Small gain and loss events are geometrically size distributed with mean 200bp, while large gain and loss events have uniform lengths between 10kbp and 50kbp. These sizes were chosen to match empirically derived estimates [9]. The results, shown in Figure 6, indicate that mauveAligner 1.3.0 falters when faced with large-scale segmental gain and loss, while progressiveMauve and TBA perform significantly better. As gain and loss rates increase in our model, the amount of orthologous sequence shared among genomes deteriorates, eventually reaching zero in the limit of infinitely high rates.

The data corresponding to these simulations are available as File S2 and File S3 for the substitution/inversion simulation and the gene gain/loss simulation, respectively.

### *Gap dribble* and the quality of long gap predictions

Gene gain and loss events manifest themselves in genome alignments as long gaps. Every predicted alignment gap implies at least one insertion or deletion of nucleotides has taken place in the history of the organisms under study. Since we would like to quantify the contribution of segmental gain and loss to the target genomes, it is imperative that predicted alignment gaps be as accurate as possible.

Current sequence alignment methods typically score pairwise alignments with an affine gap scoring scheme consisting of a gap open penalty and a gap extend penalty. In a probabilistic setting, the optimal affine-gap alignment roughly corresponds to a viterbi path alignment from a pair-HMM with a single pair of insert and delete states [52]. However, when aligning genomes which have undergone a significant amount of gene gain and loss, an excess of large gaps exists that does not fit the gap size distribution imposed by a standard global alignment pair-HMM [2]. The net result is that under the affine gap model, aligners tend to break up large gaps into a series of small gaps interspersed with short stretches of improperly aligned nucleotides. In the spirit of classifying systematic alignment errors introduced by [48], we refer to this problem as *gap dribble*, since short alignments are dribbled along the large gap. The large number of small gaps creates problems when trying to reconstruct the history of gene gain and loss events, since they imply a much greater number of insertions and deletions than actually occurred.

**Figure 7:**
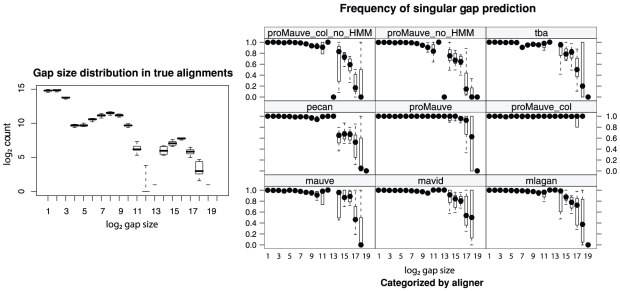
Frequency with which gaps are correctly predicted as a singular gap as a function of gap size. **Left** Average size distribution of gaps in an alignment of the nine genomes evolved at mutation rates which correspond to previous estimates for the *E. coli*, *Shigella*, and *Salmonella*. The gap size distribution was averaged over 10 simulations. **Right** Fraction of TP indel predictions that are singular TP indel predictions by true gap size. Ten replicate simulations of evolution with gene gain, gene loss, indels, and nucleotide substitution were performed and alignments were computed using each aligner. Predicted indels were classified according to the definitions given in Figure 4, namely, a singular True Positive implies the true gap is predicted as a single gap. Remaining True Positive indels have the true gap broken up into two or more predicted gaps. For each aligner, the fraction of singular predicted gaps is shown as a function of gap size. Missing points indicate a lack of TP indel predictions in that size category. All aligners do well in predicting small gaps, but large gaps present problems. Most aligners, including Pecan which uses an extra pair-HMM state to model long gaps, tend to predict long gaps as a series of short gaps interspersed with alignments of unrelated sequence. We refer to such behavior as “gap dribble.” progressiveMauve was run with default parameters (proMauve), without the Homology HMM (proMauve_no_HMM), with the option to assume genomes are collinear (proMauve_col), and finally assuming collinearity and without the HMM (proMauve_col_no_HMM).

Using our simulated evolution platform, we quantify the performance of each aligner in predicting gaps of varying size. We simulated evolution of collinear genomes (no rearrangement) that have undergone a realistic amount of gene gain and loss, corresponding to previous estimates for the rates of these events in the enterobacteria [9]. Nucleotide substitutions and indels were modeled to occur at the rate indicated by the blue asterisk in Figure 5, and gene gain and loss events were modeled to occur with twice the frequency indicated by the blue asterisk in Figure 6. Figure 7 Left gives the observed size distribution of gaps.

We then applied each aligner to the simulated genomes and measured the accuracy of gap predictions as a function of gap size. The aligners mauveAligner 1.3.0, MAVID 2.0, MLAGAN 2.0, TBA 28-02-2006, progressiveMauve, and Pecan v0.7 were tested. Pecan v0.7 is a new aligner that has been demonstrated to have excellent performance [33], [45] by virtue of using probabilistic consistency during the anchoring process. Moreover, Pecan v0.7 uses a pair-HMM with an extra gap state specifically designed to model long indels. The reconstructed alignments were scored against the true alignments and results for ten replicates were recorded.

The right side of Figure 7 shows the quality of each aligner's indel predictions as a function of the true gap size. Shown is the frequency with which gaps of a particular size are predicted as a single gap (singular TP) instead of a string of smaller gaps with interspersed alignments of non-homologous sequence (nonsingular TP). From the figure, it is obvious that aligners which use an affine gap penalty tend to perform poorly in predicting large gaps. Somewhat surprisingly, the pair-HMM with an extra gap state used by Pecan to model long indels still yields poor predictions of long gaps, although sensitivity is quite good (not shown). progressiveMauve appears to perform well at all gap sizes, especially when the aligner is told to explicitly assume the genomes are collinear (proMauve_col). To determine whether progressiveMauve's performance results from its anchoring algorithm or use of the Homology HMM to reject alignments of unrelated sequence, we also tested progressiveMauve without the Homology HMM, shown as panels proMauve_no_HMM and proMauve_col_no_HMM. Without the Homology HMM progressiveMauve yields inferior results, indicating that the Homology HMM does indeed address the problem of gap dribble. The Homology HMM functionality of progressiveMauve is available via command-line interface, so it is possible to apply it to any alignment in the XMFA format.

## Discussion

progressiveMauve excels at aligning rearranged genomes with different gene content. The so-generated positional homology alignments enable a wide variety of downstream research. Here we illustrate some applications with an alignment of 23 complete *E. coli*, *Shigella* and *Salmonella* genomes. The alignment can be used to characterize the shared (core) and total (pan-genome) amount of sequence found in these species. The alignment can also be used to extract variable sites for more traditional phylogenetic analyses. progressiveMauve identifies and aligns both conserved regulatory regions and hypervariable intergenic regions.

The progressiveMauve alignment of twenty-three *E. coli*, *Shigella*, and *Salmonella* genomes reveals a core genome of 2,675 segments conserved among all taxa, which account for an average of 2.46 Mbp of each genome. Between the core segments lie regions conserved among subsets of taxa and regions unique to individual genomes. By counting each core, unique, and subset segment exactly once, one constructs a total pan-genome that includes genes and intergenic regions alike. The 23 genomes have a pan-genome of 15.2 Mbp, approximately three times that of a single strain, indicating a tremendous degree of variability in both genic and intergenic content.

**Figure 8:**
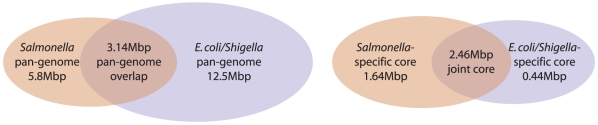
Venn diagram of the pan-genome (left) and core genome (right) of *E. coli/Shigella* and *S. enterica*.

We now focus specifically on the genomic variation among the *E. coli* and *Shigella*. *Shigella* spp. are widely recognized as *E. coli* based on phylogenetic analyses [53] and genome comparisons [54], though the original phenotypically derived taxonomy persists. We will refer to them collectively as *E. coli/Shigella*. Similarly, taxonomic revisions of *Salmonella*, have collapsed almost all strains into a single species: *S. enterica*. Thus, we are examining the structure of the pan and core genomes of two sister species, *E. coli/Shigella* and *Salmonella*. The 16 *E. coli/Shigella* strains have a pan-genome of 12.5 Mbp and core of 2.9 Mbp, while the seven *S. enterica* serovars have a pan-genome of 5.8 Mbp and a core genome of 4.1 Mbp. The intersection of the core genomes is the joint core, while the union of the pan-genomes is the combined pan-genome, shown in Figure 8. Note that the intersection of pan-genomes is 580 kb larger than the joint core. This counter-intuitive situation arises when components of the core-genome of one group are found in some, but not all members of the other species. In this instance, 220 kb can be attributed to losses of genes in *Shigella* strains that are otherwise conserved among all *E. coli* and *Salmonella*. A more detailed dissection of the patterns of gene gain and loss in the *E. coli* and *Shigella* based on progressiveMauve alignments has been given elsewhere [55].

In comparison with the core- and pan-genome sizes estimated using intact protein coding genes, we expect our core-genome estimates to be somewhat larger and the pan-genome smaller, because our method can include any positionally homologous segment and not just intact genes. In 17 *E. coli* isolates, Rasko et al. found 2200 conserved genes and 13,000 genes in the pan-genome [56]. The average gene size in *E. coli* is slightly less than 1000nt. Based on the average gene length, our method finds an additional 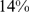 of the average genome to be part of the core and a reduction in the pan-genome size of 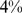 in a similar number of genomes. However, our study includes *Shigella* which was not included in the protein-based study [56], so organism sampling may also contribute to differences in core- and pan-genome sizes.

### Inference of genome rearrangement history

progressiveMauve alignments also make an excellent starting point for analysis of genome rearrangement patterns. Genome rearrangement is known to occur via a multitude of mutational forces, including inversion, transposition, and duplication/loss, and is especially prominent in bacterial pathogens. Methods already exist to infer inversion histories among pairs of genomes [57], [58] and multiple genomes [59], [60]. More general models to account for multiple chromosomes and multi-break rearrangements have also been developed [61]–[63], although not yet in the Bayesian phylogenetic context.

Most genome rearrangement history inference methods do not also infer gene gain and loss, but instead assume that gene content across genomes is equal. When gene content is nearly equal, current models can use a multiple genome alignment to infer patterns of genome rearrangement [64]. However, equal gene content has proved to be the exception rather than the rule. Despite that, a progressiveMauve alignment with differential content can be trivially reduced to contain only segments conserved among all taxa of interest, yielding a signed gene-order permutation matrix that is suitable for current genome rearrangement inference software.

A further avenue of genome rearrangement inference would be to combine the positional homology information in a progressiveMauve genome alignment with the repeat family information available from general local-multiple alignment programs such as Repeatoire [34]. One could then infer repeat-annotated phylogenetic trees using genome arrangement information [65]. Such an approach might be especially appropriate for bacteria where homologous recombination among repeats appears to play a major role in genome rearrangement.

### Alignment visualization

Genome alignments are large and complex entities that are not usually suitable for direct interpretation. Genome comparison browsers such as the UCSC browser [66], VISTA [67], and others have proven invaluable as tools to facilitate understanding of whole genome alignments. To aid in use of progressiveMauve alignments, we have developed an interactive visualization program that can present a complex alignment in a meaningful and easily understandable visual paradigm.

**Figure 9:**
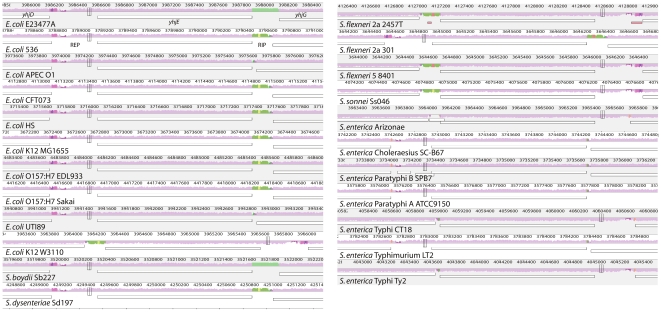
A Mauve visualization of the hypervariable intergenic regions surrounding *yhdE*. Each genome is laid out in a horizontal track, with annotated coding regions shown as white boxes. A colored similarity plot is shown for each genome, the height of which is proportional to the level of sequence identity in that region. When the similarity plot points downward it indicates an alignment to the reverse strand of the genome. Colors in the similarity plot indicate the combination of organisms containing a particular segment of the genome. Segments colored pink/mauve are conserved among all organisms, while purple segments are conserved in everything but *Salmonella*, and segments colored in olive green are conserved among non-uropathogenic *E. coli*. The visualization system is interactive and written in Java, and works on all computers supporting Java 1.4 or later.

The visualization system illustrates three major aspects of genome evolution: genome rearrangement, patterns of segmental gain and loss, and the extent of local conservation of nucleotide sequences. Figure 9 illustrates the latter two aspects in a visualization of the 23-way alignment of *E. coli*, *Shigella*, and *Salmonella*.

Figure 9 shows the region surrounding the *yhjE* gene, which encodes a product in the Major Facilitator Superfamily of transporters. *yhjE* is flanked by *yhjD* to the left, and *yhjG* to the right. The intergenic regions between these three genes are hypervariable (as indicated by the variety of colors in Figure 9) and have been subject to multiple insertion and deletion events. The hypervariable nature of the regions surrounding *yhjE* may not be surprising, because it harbors a REP element to the left, and a RIP element to the right. REP elements contain a series of two or more 35-bp palindromic repeats and are known for a variety of functions, including the binding of DNA gyrase and PolI, and as mRNA anti-decay hairpins or rho-dependent attenuators. RIP elements are a specialized form of REP elements that contain an IHF binding site [68], and this particular RIP also contains a REPt transcription terminator sequence. IHF is a global transcriptional regulator in *E. coli*. Repeat elements are known to be unstable in bacterial genomes [47].

Interestingly, the patterns of insertion/deletion in the intergenic regions surrounding *yhjE* do not follow the expected taxonomic patterns, suggesting instead that recombination among strains has taken place. The RIP region present to the right of *yhjE* in most *E. coli* has been replaced with an unrelated sequence in *E. coli* E23477A and *S. boydii* (shown as turquoise in Figure 9), but not in *S. sonnei*. Those three strains form a clade in the *E. coli*/*Shigella* taxonomy [55] with *E. coli* E23477A branching first, so convergent evolution must have occurred here.

The pattern of intergenic variability surrounding *yhjE* suggests potential regulatory divergence, a much studied evolutionary mechanism in eukaryotes often overlooked in microbial research. The *yhjE* locus is by no means the only region harboring intergenic variablity; a screen of the 23-way alignment identifies 102 other strictly intergenic regions with similarly variable conservation patterns.

### Scalability to large genomes and many genomes

The algorithm is complex and many factors contribute to the overall asymptotic time complexity and running time of the algorithm in practice. The novel sum-of-pairs anchoring heuristic used by progressiveMauve is at least 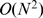 in time complexity, since it requires evaluation of LCBs among pairs of genomes. We find that alignments of 5 genomes averaging 5Mbp in size can be computed in under an hour on a single computer, 20 genomes of the same size can be computed about 24 hours using 4Gb of memory, and alignments of 40 *Escherichia* and *Shigella* genomes of 5Mbp can be computed in 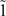
70 compute hours with 8GB RAM. The main contributing factors to wall-clock runtime are number of genomes and divergence of those genomes, with higher values of each causing fast growth in both memory and time requirements. Alignment of larger genomes is feasible; pairwise alignment of *Drosophila melanogaster* and *Drosophila yakuba* requires less than 3 hours on a single computer, while alignment of the human and mouse genomes requires 90GB RAM and about 32 compute hours. The human/mouse alignments and the alignment of 40 *E. coli* and *Shigella* genomes are available for download from http://biotorrents.net [69].

We note that many parts of the algorithm are independent and would be amenable to parallelization, however the current release (version 2.3.1) runs in serial mode only.

### Gene duplications: *glocal* versus *positional homology* alignment

As described above, progressiveMauve generates *positional homology* multiple genome alignments. These alignments differ substantially from *glocal* genome alignments, wherein all copies of a repetitive gene family may become aligned to each other. In our view, the *positional homology* alignment is most useful for comparison of closely related microbes for several reasons. First, the contribution of whole genome duplication and segmental duplication in most bacteria and archaea is thought to be small. Second, even though large tandemly repeated segments are generated very frequently in microbes and may be a fundamental process in adaptive evolution, they are extremely unstable and generally do not persist over long periods of time [70]. For this reason, a method which optimizes long collinear regions for alignment generally will identify and align the correct positional homolog. The so-aligned positional homologs will often be orthologs, or in the case of lateral gene transfer, they will be xenologs. The positional homology alignment facilitates downstream alignment tasks such as phylogenetic inference of nucleotide substitution, phylogenetic inference of gene gain and loss [55], phylogenetic inference of rearrangement [64], and even inference of homologous recombination-induced lateral gene transfer [71].

The main disadvantage of a positional homology alignment is that by itself it does not provide a suitable basis for inference of within-genome gene conversion or inference of gene duplication histories. Especially in scenarios where some of the input genomes have undergone whole-genome duplication, the *positional homology* alignment method may fail to align many target regions. A *glocal* alignment by itself can be used for such purposes. Inference tools to reconstruct genome evolution based on *glocal*-type alignments have begun to appear [72], and some have even begun to incorporate nucleotide-level evolutionary models [73]. Still, a model that incorporates both nucleotide and genome arrangement evolution remains to be implemented.

One major shortcoming of our simulation study is that it does not explicitly model gene duplication. Even though duplicated material can be indirectly created by repeated gain of the same region, our simulation platform does not quantify the frequency with which that happens. Therefore we are unable to objectively characterize the accuracy of alignment methods at particular rates of simulated gene duplication. However, by manually inspecting alignments of multi-gene families in the Mauve alignment viewer, we have observed that the progressiveMauve algorithm aligns the positional homolog in many cases where our previous mauveAligner algorithm failed to do so. progressiveMauve is an improvement with respect to alignment of positional homologs in multi-gene families, but a full characterization of limitations with respect to gene duplication remains for future work.

### Conclusions

We have presented a novel multiple genome alignment heuristic that extends our previous approach by aligning regions conserved in subsets of genomes. which demonstrates a substantial accuracy improvement on simulated datasets. Key features of the approach are an anchor scoring function that penalizes alignment anchoring in repetitive regions of the genome and penalizes genomic rearrangement. Use of a Sum-of-pairs approach enables robust scoring of genomes that have undergone gene gain, loss and rearrangement—a scenario not addressed by our previous alignment method.

Future efforts to improve genome alignment may explicitly incorporate models of evolutionary distance into alignment scoring process [74]. Multiple alignment methods based on probabilistic consistency have demonstrated great promise in the context of amino-acid alignment [75] and aligning collinear genomic regions [33], and in principle, could be extended to genome alignment with rearrangement. Other recent efforts have developed fast approximations to statistical alignment [51] and such methods will surely factor into future approaches to align genomes with rearrangement.

No method reconstructs error-free genome alignments, and any particular alignment is likely to contain errors that can substantially influence downstream inference. However, methods to estimate the confidence in aligned columns are under continuing development [48], [51]. Downstream inference methods that can explicitly cope with the inherent uncertainty in reconstructed alignments will be crucial for continued advances in comparative genomics.

## Supporting Information

Table S1 A listing of bacterial strains and accession numbers included in the 23-way genome alignment.
(0.03 MB PDF)

Figure S1 Accuracy results for MLAGAN 2.0 and MAVID 2.0 on genomes simulated with rearrangement and gene flux. Neither software was designed to handle such cases directly.
(0.06 MB PDF)

File S1 Accuracy results for nt substitution and indels. Evolution was simulated along a fixed 9-taxon tree encoded as a newick string in the file simujobparams.pm. A range of substitution rates were simulated from 0 substitutions per site to about 0.9 substitutions per site among the most distant taxa in the fixed tree. In conjunction, a range of indel rates were also simulated, up to about 0.18 indels per site among the most distant taxa. For each simulation, the true alignment is recorded along with the set of evolved sequences. Aligners are then run to reconstruct the true alignment. The program scoreAlignment2 is used to calculate various accuracy metrics on the reconstructed alignments. These accuracy metrics include sensitivity and positive predictive value when for aligning homologous nucleotides, along with similar metrics for identifying indels. The scoreAlignment2 program also generates an indel boundary report, although it in not included in this archive due to size constraints. The indel boundary report records, for every indel in the true alignment, how close the boundaries of the predicted indel were in the alignment calculated by an aligner. The report contains the size of the true and predicted indels, so that one can generate summaries of indel accuracy stratified by size. Note: this archive must be decompressed with 7-zip first and then tar. This archive contains results of accuracy evaluations on each aligner program. The simulated alignments themselves are not contained in the archive, as they would be far too space-consuming. Instead, each subdirectory contains the summary of the accuracy tests, along with all simulated evolution parameters used, and importantly, the random seed used for simulation so that the each alignment dataset can be reconstructed. To reconstruct the original alignments, one must also obtain a few freely-available programs and scripts, as described on http://asap.ahabs.wisc.edu/mauve-aligner/mauve-developer-guide/evaluating-alignment-quality-and-stress-testing-the-aligner.html. Subdirectories are named first according to the aligner tested, e.g., mauve =  = mauveAligner, promauve =  = progressiveMauve, mavid =  = Mavid 2.0, mlagan =  = MLAGAN 2.0, tba =  = TBA 2006-02-28. The remaining portion of each subdirectory name indicates the type of experiment performed. ntsub_indel is for collinear genomes simulated with increasing rates of substitution and indels. ntsub_inv is for genomes simulated with increasing rates of inversion rearrangements and nucleotide substitution. geneflux is for genomes simulated with increasing rates of small- and large-scale gain and loss (flux) of genes. Note that the full indel boundary accuracy results have also been omitted, as they include several numerical values for every indel of every simulated alignment and were therefore too space-consuming. They can of course be regenerated using the simulation scripts and the random seeds contained in this archive.
(5.74 MB TAR)

File S2 Accuracy results for gene gain and loss (flux). Evolution was simulated along a fixed 9-taxon tree encoded as a newick string in the file simujobparams.pm. Nucleotide substitution and indel rates were fixed so that the most distant taxa would have divergence similar to *E. coli* and *Salmonella*. Large gene gain and loss events were simulated with rates giving 0 to 150 events along the path connecting the most distant taxa in the fixed tree. In conjunction, small gene gain and loss events were also simulated, ranging from 0 up to about 10000 events among the most distant taxa. For each simulation, the true alignment is recorded along with the set of evolved sequences. Aligners are then run to reconstruct the true alignment. The program scoreAlignment2 is used to calculate various accuracy metrics on the reconstructed alignments. These accuracy metrics include sensitivity and positive predictive value when for aligning homologous nucleotides, along with similar metrics for identifying indels. The scoreAlignment2 program also generates an indel boundary report, although it in not included in this archive due to size constraints. The indel boundary report records, for every indel in the true alignment, how close the boundaries of the predicted indel were in the alignment calculated by an aligner. The report contains the size of the true and predicted indels, so that one can generate summaries of indel accuracy stratified by size. Note: this archive must be decompressed with 7-zip first and then tar. This archive contains results of accuracy evaluations on each aligner program. The simulated alignments themselves are not contained in the archive, as they would be far too space-consuming. Instead, each subdirectory contains the summary of the accuracy tests, along with all simulated evolution parameters used, and importantly, the random seed used for simulation so that the each alignment dataset can be reconstructed. To reconstruct the original alignments, one must also obtain a few freely-available programs and scripts, as described on http://asap.ahabs.wisc.edu/mauve-aligner/mauve-developer-guide/evaluating-alignment-quality-and-stress-testing-the-aligner.html. Subdirectories are named first according to the aligner tested, e.g., mauve =  = mauveAligner, promauve =  = progressiveMauve, mavid =  = Mavid 2.0, mlagan =  = MLAGAN 2.0, tba =  = TBA 2006-02-28. The remaining portion of each subdirectory name indicates the type of experiment performed. ntsub_indel is for collinear genomes simulated with increasing rates of substitution and indels. ntsub_inv is for genomes simulated with increasing rates of inversion rearrangements and nucleotide substitution. geneflux is for genomes simulated with increasing rates of small- and large-scale gain and loss (flux) of genes. Note that the full indel boundary accuracy results have also been omitted, as they include several numerical values for every indel of every simulated alignment and were therefore too space-consuming. They can of course be regenerated using the simulation scripts and the random seeds contained in this archive.
(7.73 MB TAR)

File S3 Accuracy results for inversion and nucleotide substitution simulations. Evolution was simulated along a fixed 9-taxon tree encoded as a newick string in the file simujobparams.pm. Indel rates were fixed to a low value so that the most distant taxa would have indels similar to *E. coli* and *Salmonella*. Inversion events were simulated with rates giving 0 to about 1400 events along the path connecting the most distant taxa in the fixed tree. In conjunction, nucleotide substitution events were also simulated, ranging from 0 up to about 0.9 substitutions per site among the most distant taxa. For each simulation, the true alignment is recorded along with the set of evolved sequences. Aligners are then run to reconstruct the true alignment. The program scoreAlignment2 is used to calculate various accuracy metrics on the reconstructed alignments. These accuracy metrics include sensitivity and positive predictive value when for aligning homologous nucleotides, along with similar metrics for identifying indels. The scoreAlignment2 program also generates an indel boundary report, although it in not included in this archive due to size constraints. The indel boundary report records, for every indel in the true alignment, how close the boundaries of the predicted indel were in the alignment calculated by an aligner. The report contains the size of the true and predicted indels, so that one can generate summaries of indel accuracy stratified by size. The average distance between a true rearrangement breakpoint and a predicted rearrangement breakpoint is also reported by scoreAlignment2. Note: this archive must be decompressed with 7-zip first and then tar. This archive contains results of accuracy evaluations on each aligner program. The simulated alignments themselves are not contained in the archive, as they would be far too space-consuming. Instead, each subdirectory contains the summary of the accuracy tests, along with all simulated evolution parameters used, and importantly, the random seed used for simulation so that the each alignment dataset can be reconstructed. To reconstruct the original alignments, one must also obtain a few freely-available programs and scripts, as described on http://asap.ahabs.wisc.edu/mauve-aligner/mauve-developer-guide/evaluating-alignment-quality-and-stress-testing-the-aligner.html. Subdirectories are named first according to the aligner tested, e.g., mauve =  = mauveAligner, promauve =  = progressiveMauve, mavid =  = Mavid 2.0, mlagan =  = MLAGAN 2.0, tba =  = TBA 2006-02-28. The remaining portion of each subdirectory name indicates the type of experiment performed. ntsub_indel is for collinear genomes simulated with increasing rates of substitution and indels. ntsub_inv is for genomes simulated with increasing rates of inversion rearrangements and nucleotide substitution. geneflux is for genomes simulated with increasing rates of small- and large-scale gain and loss (flux) of genes. Note that the full indel boundary accuracy results have also been omitted, as they include several numerical values for every indel of every simulated alignment and were therefore too space-consuming. They can of course be regenerated using the simulation scripts and the random seeds contained in this archive.
(9.16 MB TAR)
