# Supplementary material for: progressiveMauve: Multiple Genome Alignment with Gene Gain, Loss and Rearrangement
Source: PLoS One. 2010 Jun 25;5(6):e11147. doi: 10.1371/journal.pone.0011147 (PMC2892488; doi:10.1371/journal.pone.0011147)
Supplement: Table S1 — A listing of bacterial strains and accession numbers included in the 23-way genome alignment. (0.03 MB PDF) [file pone.0011147.s001.pdf]

Organism	Genome size w/Plasmids	Accession
E. coli K-12 MG1655	4,654,221	U00096
E. coli K-12 W3110	4,646,332	AP009048
E. coli HS	4,643,538	AAJY00000000
E. coli O157:H7 EDL933	5,623,806	AE005174
E. coli O157:H7 Sakai	5,594,477	BA000007
E. coli E24377A	4,980,187	AAJZ00000000
E. coli CFT073 (UPEC)	5,231,428	AE014075
E. coli UTI89 (UPEC)	5,179,971	CP000243
E. coli APEC O1	5,082,025	CP000468
E. coli 536 (UPEC)	4,938,920	CP000247
Shigella boydii Sb227	4,646,520	CP000036
Shigella flexneri 2a 2457T	4,988,914	AE014073
Shigella flexneri 2a 301	4,828,821	AE005674
Shigella flexneri 5 8401	4,574,284	CP000266
Shigella dysenteriae Sd197	4,551,958	CP000034
Shigella sonnei Ss046	5,039,661	CP000038
Salmonella enterica Choleraesius B67	4,944,000	AE017220
Salmonella enterica Typhi Ty2	4,791,961	AE014613
Salmonella enterica Typhi CT18	5,133,713	AL513382
Salmonella enterica Typhimurium LT2	4,951,371	AE006468
Salmonella enterica Paratyphi A ATCC9150	4,585,229	CP000026
Salmonella enterica Arizonae	4,600,800	NC_010067
Salmonella enterica Paratyphi B SPB7	4,858,887	NC_010102

Table 1: Twenty-three publicly-available, finished genome sequences from the genera *Salmonella*, *Escherichia*, and *Shigella* form our target set for multiple genome alignment.
