# Supplementary material for: progressiveMauve: Multiple Genome Alignment with Gene Gain, Loss and Rearrangement
Source: PLoS One. 2010 Jun 25;5(6):e11147. doi: 10.1371/journal.pone.0011147 (PMC2892488; doi:10.1371/journal.pone.0011147)
Supplement: Figure S1 — Accuracy results for MLAGAN 2.0 and MAVID 2.0 on genomes simulated with rearrangement and gene flux. Neither software was designed to handle such cases directly. (0.06 MB PDF) [file pone.0011147.s002.pdf]

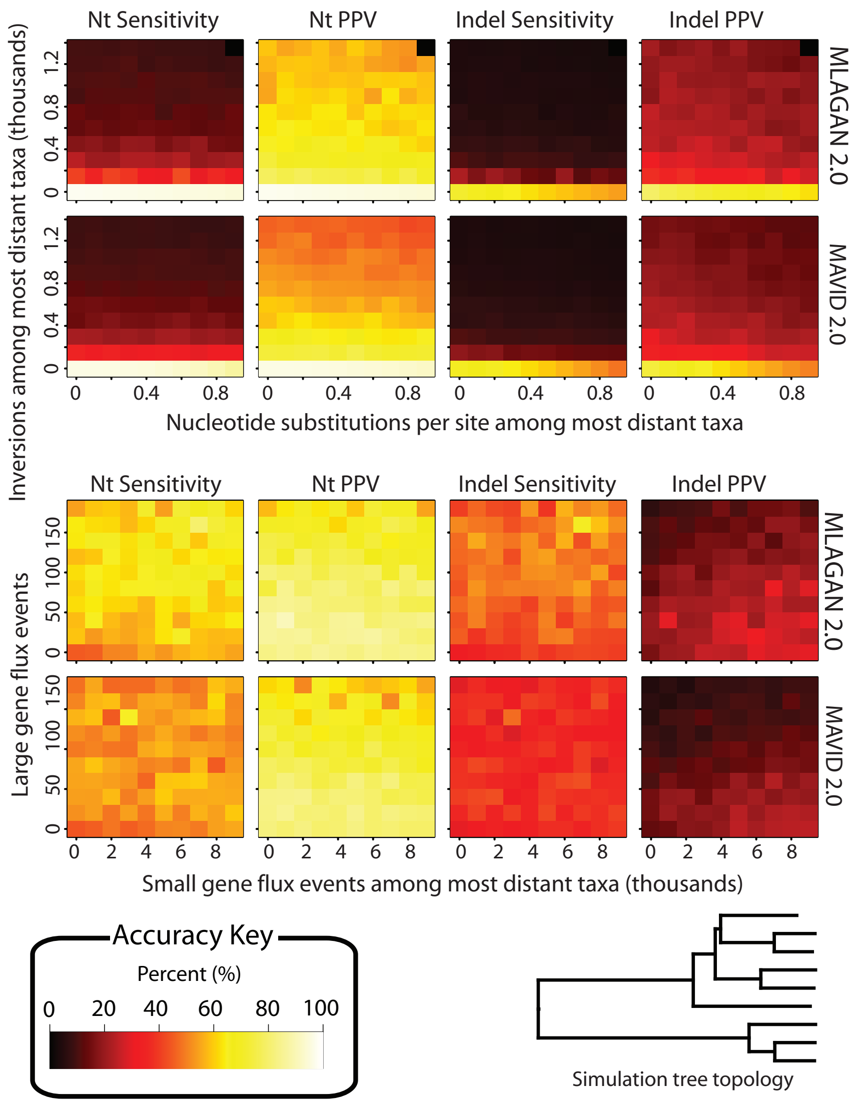

Figure 1: The performance of MLAGAN 2.0 and MAVID 2.0 on genomes evolved with inversions, gene gain, and gene loss corresponding to Figure 4 in the main text. Neither MLAGAN nor MAVID were designed to align genomes with rearrangement and differential content; this figure demonstrates the decay in alignment quality when such forces are present in the data but not modeled by the aligner. The black dot in the upper right corner of the MLAGAN results indicates that MLAGAN did not finish alignments at the highest combination of inversion and substitution rate within the allotted 10 hour limit.
